# Supplementary material for: Effects of biobanding on training loads and technical performance of young football players
Source: PLoS One. 2025 Feb 13;20(2):e0317432. doi: 10.1371/journal.pone.0317432 (PMC11824955; doi:10.1371/journal.pone.0317432)
Supplement: Attachment — Submitted filename: Response Letter_PlosOne.docx [file pone.0317432.s001.docx]

July 26, 2024

RE: Revised version of the manuscript - PONE-D-24-11339 “EFFECTS OF BIOBANDING ON TRAINING LOADS AND TECHNICAL PERFORMANCE OF YOUNG FOOTBALL PLAYERS”.

PLOS ONE”

Dear Editor-in-Chief,

First of all, we appreciate the time devoted to evaluating our manuscript, helping us to craft hopefully an improved version. We are pleased to clarify your concerns, which we believe will improve the impact and quality of our work. We have made a concerted attempt to systematically address the specific concerns raised for this revision. We have highlighted the alterations in the manuscript in yellow for convenience. Your constructive and insightful points have improved selected areas of the manuscript significantly. We believe that the manuscript has been significantly improved based on your insightful comments and we hope that based on these modifications, the manuscript is now closer to acceptance.

**Reviewer Comments:**

**Reviewer #1:** Dear authors, we have found that there may be the following problems in your paper, which need your explanation or appropriate modification:

1. Please explain how subjects are recruited and screened.

**RESPONSE:** Thank you for this comment. The young athletes were recruited based on convenience, as they were already participating in the youth divisions of the club where the research was conducted. This approach is justified by the nature of the study, which required that all young athletes meet the primary inclusion criterion of being trained under the same conditions, i.e., the same training duration, number of weekly training sessions, and external training load. This information was included in the text as follows:

*Twenty young footballers (11.8 ± 1.15 years), volunteered for this study. They were recruited based on convenience to meet the inclusion criteria of the study: (A) participation in the under-13 category, (B) training with the group for at least six months, and (C) having attended at least 80% of the training sessions during this period.*

2. Lower extremity strength usually increases with the age of adolescents, and the CMJ of adolescent athletes who are older and participate in sports for a longer time is usually higher than that of younger athletes. Please explain why the older PHV group in your study had significantly lower CMJ than the pre-PHV group of athletes.

**RESPONSE:** We appreciate the reviewer's comments. Please note that CMJ differences were not significant between groups (p=0.420). This condition is supported by the studies of Jones et al. (2020)^[1]^ that showed no significant difference for the Eccentric knee flexor in Under-11, 12 and 13 groups and Moreira et al. (2013)^[2]^ which also did not indicate significant differences between groups that had not surpassed PHV.

2.1 If possible, please provide raw data of the subject's Age, yPHV, Height, Body mass, CMJ, as these will affect their internal and external load and SSGs performance, which is one of the factors that must be considered for grouping.

**RESPONSE:** Thank you for this comment. The aim of our study was to investigate weather grouping young players according to somatic maturation would lead to a better training load profile during small-sided games. Therefore, as per experimental manipulation differences in maturational levels were significant between groups (-2.2 yPHV vs. -0.89 yPHV). Furthermore, the method used to determine maturational considered weight, height, and age of the participants. As such, we understand that considering all variables will result in excessive control of variables and will mischaracterize the biobanding strategy under scrutiny in our study.

3. Data may be wrong. In Table 2, the age, height, weight and other data of athletes in PHV group and pre-PHV group are different from the mean value of all athletes after calculation. For example, adding the CMJ data for athletes in the PHV and pre-PHV groups yielded a CMJ mean (25.315) that was different from the CMJ mean for all athletes (25.03).

**RESPONSE:** We appreciate the careful review of our study. However, the presented data is not wrong. The average of the whole group for CMJ was indeed 25.03. If you take the average of two subgroups and calculate an average of them both the value will be slightly different than the whole group average.

4. In the table with significant differences between the two groups of data, please report the specific P-value and other relevant index values.

**RESPONSE:** We appreciate the reviewer's comments. Please see the text for exact p-values. Inserting them again in the tables will lead to unnecessary duplication of information.

5. Since all the athletes in your study were under the age of 13, the differences in maturity were not significant enough. Differences in body weight, training level, and field position of the subjects in the study can also interfere with the results, and it should be explained how to control for these variables.

**RESPONSE:** Thank you for this comment. Firstly, as per experimental manipulation we stress that the maturation level between groups was significant. The PHV group were at PHV with an average maturation of -0.89 ± 0.36 yPHV and the pre-PHV were at -2.2 ± 0.67 yPHV. These groups were significantly different regarding their maturation levels. A possible limitation of our study is the lack of a post-PHV group. This information has been discussed in our submitted article (please see page 12)

As discussed in item 2.2 of this review, we understand that body weight is impacted by maturation and that is the reason why we conducted the study. Further control for this variable is beyond the scope of our article.

Moreover, training level is an important consideration and we tried to minimize by selecting a group of participants, who albeit of different ages, were participating in the same type of training for at least 6 months.

Regarding players positions, participants did not have clearly defined roles in the squad. This information was inserted in the text as follow:

*Considering the category and the age groups included the positions on the field have not yet been clearly defined.*

**Reviewer #2:** I would like to thank the authors for this study examining an interesting and relevant issue regarding maturational differences and the effects of biobanding on technical performance. Please find my comments below.

**RESPONSE:** Thank you for your kind comment.

6. One of my major concerns is the presentation of the research data. More specifically, in addition to the results presented in the manuscript, it would be useful to see also the results for each session separately. By doing this, a clearer view on data variability and consistency according to condition (BIO or CA) could be examined.

**RESPONSE:** We appreciate the reviewer’s comments. Using two SSGs in each condition is a strength of our investigation. This is because playing twice in the same condition would increase certainty in the observed findings. Furthermore, to model the impact of the condition, maturation and interaction we used a linear mixed-model approach with a repeated design to include both matches with each participant with 4 observations in the model (80 observation). From there, we inserted random effects for participants. We are confident that this approach is the best to deal with the repeated design rather than averaging the two matches into one score. Presenting both sessions separately rather than modeling the date would lead to uncertainty.

7. Also, a clear explanation is needed on the use of the external load (as applied here) in the examined age range. Does it really reflect true load at such young age? Why changes in acceleration were preferred over other metrics (e.g. total distance, running speed)? And how is it connected to the internal load (HR)? Please provide a strong theoretical background supported by appropriate references.

**RESPONSE:** Thank you for this suggestion. PlayerLoad was used as a measure of external load because it is a more comprehensive metric, quantifying the total movement performed by players across three axes (horizontal, vertical, and lateral). Other studies have used the same method with athletes of similar age groups (Castellano et al, 2016^[3]^ and Silva et al, 2024^[4]^) and with young athletes [17 ± 0.9 years (Denmark et al, 2021^[5]^)] demonstrating its effectiveness in this population. Additionally, PlayerLoad is an indicator that correlates with the session RPE method [(r = .51, p < .001) (Silva et al, 2024^4^)] and Edwar’s method (Askow et al, 2021^[6]^) (Casamichana et al, 2012^[7]^). This information has been included in the manuscript, please see page 06.

8. The study includes players ranging from 10 to 13 years. How reasonable is to have children of 3 years difference in CA grouped together? Please, justify the selection of this age range.

**RESPONSE:** Thank you for this observation. Our investigation was conducted inside a football club where children unde-13 trained together. This included children aged 10-13 years old. This age range in training and competition is common in our country, especially in football. The age range inside the under-13 category is one of the rationales why we believe biobanding should be used for training.

Abstract
9. Please include a short description on the rationale of the study. EM Abbreviation is not explained in the abstract. The conclusions seem to repeat the results, please provide conclusions and the practical relevance of the results in the last paragraph.

**RESPONSE**: Thank you for this suggestion. We have now inserted the short rationale in the abstract as follow:

*Background: In adolescent sports, grouping by chronological age can advantage athletes born early in the year due to maturation differences. Early maturing athletes often achieve greater performance gains, are perceived as more talented, and receive more specialized training and workloads. This study aimed to assess the effects of biobanding on internal and external training loads, as well as technical performance during small-sided games (SSGs).*

Introduction
10. In general, introduction is well-written. Again, a justification of the indicators used to assess external load in small- sided games would contribute to better understand the results. In its present form it is not convincing that what we measure is what we want to know.

**RESPONSE:** Thank you for this suggestion. We have now inserted a paragraph in the introduction as follow:

*Thus, using external training load analysis instruments, such as the PlayerLoad method—which quantifies total movement across three axes (horizontal, vertical, and lateral)—along with measuring session-RPE and training impulse (TRIMP), enables the assessment of internal training load using both objective and subjective data (Casamichana et al. 2012). This approach allows for a comprehensive analysis of the impact of external load on the player. By combining external load values with internal load measurements, we can evaluate a player's ability to handle the training session (Bourdon et al. 2017). On the other hand, analyzing technical parameters of the game can provide valuable insights into a player's involvement, depending on their maturational status. Biobanding, in particular, can create more opportunities for late-maturing players to utilize their technical competence effectively. (Lüdin et al. 2022).*

11. When using in-text citations please reconsider according to: Author et al. (1) found no effects on…

**RESPONSE:** Thank you for your observation. Done accordingly.

12. Ln 68. This sentence seems not to be completed: reduce discrepancies caused by…???

**RESPONSE:** Thank you for your observation. This phrase has been removed from the text.

13. Ln 76-78. This sentence is not clear, please consider rephrasing. What do you mean by morpho functional adaptations?

**RESPONSE:** Thank you for this comment. The sentence has been rewritten as follow:

*In the context of football training, using methods that facilitate both morphological and functional adaptations and the acquisition of sport-specific skills may prove superior, especially in early years of athletic development*

14. Ln 78. Consider starting a new sentence here (In this way,…)

**RESPONSE:** Done accordingly.

Methods
15. It is not clear how grouping based on CA was performed (detailed explanation is needed). Again, I don’t think that is usual to group boys in this age range (10 to 13 years) together. What maturational differences we may expect in 10-year-old boys? It is easy to think that maturation does not affect performance, considering that at this age boys are still well before their APHV. Otherwise, please explain providing convincing references.

**RESPONSE:** Thank you for this comment. Regarding the how grouping based on CA was performed, the explanation is inserted in the text as follow:

*1) CA - SSGs with players divided according to their chronological age (i.e., Randomized teams without the use of biobanding). The CA group was randomized while ensuring that all age ranges were similarly represented in each of the 4 teams formed. Each team included players aged 10, 11, 12 and 13 years old, ensuring balanced age distribution across all teams.*

Furthermore, the authors understand the reviewer's concern; however, as explained in item 8, only one player was younger than those normally seen in the under-13 category. In the authors' opinion, the possible maturational difference of this athlete could increase the likelihood of differences in results between the groups (BIO and CA). However, since the athlete played in both conditions, the possible differences (chronological and biological) would be equally distributed between the groups, which would not interfere with the final result. Analyses without this athlete produced results very similar to those presented in the study.

The authors agree with the reviewer that maturational status does not affect performance in the years before PHV^[8],^^[9]^, likewise, the majority of the results from this research support this statement. Most of the athletes had not reached or surpassed PHV, and this was decisive for the final research result.

16. Data of the different conditions were summed/averaged per game? Please, include this information in the manuscript.

**RESPONSE:** Thanks for this comment. Our statistical model handles repeated data in the models. As such, we inserted both games in the models and adjusted per participant accordingly. Please see the statistical section for details.

17. Ln 190. A reference is needed here.

**RESPONSE:** Thanks for this comment. Done accordingly (DOI: 10.1039/C5JA00380F)

18. Considering the low sample size effect size results seem necessary to confirm any meaningful differences. Please add to the statistical analysis a description of the inter-session reliability (ICC calculation), which is reported in the results.

**RESPONSE:** We have presented the estimated effects from the linear mixed-models. We are unaware of any other effect size estimate for the statistical approached used in our study. If the reviewer has a different understanding of effect and could point us to where we could find reliable effect sizes for linear mixed-models we would appreciate. However, please note that the presented data in Table 4 are estimated effects (and associated uncertainty) from the linear mixed models. These can be used as effects estimates. For example, it was observed that for external training load the estimated effect for the chronological condition was -26.71 AU compared to BIO.

Results
19. Differences in maturational status may be also explained with the differences in their CA. PHV players in PHV group were 1.5 year older than those in the pre-PHV group. In this sense we cannot speak a bout player of the same CA, but of different maturational status.

**RESPONSE:** Thanks for this comment. In fact, the reviewer is correct; however, we did not specifically address players of the same CA. the CA group refers to the under-13 category (ranged 10-13 years old). This information was insert in the *Design section* as follow:

*This experimental study involved participants performing SSGs under two different conditions: 1) CA - SSGs with players divided according to their chronological age (i.e., Randomized teams without the use of biobanding). The CA group was randomized while ensuring that all age ranges were similarly represented in each of the 4 teams formed. Each team included players aged 10, 11, 12 and 13 years old, ensuring balanced age distribution across all teams.*

Specifically, in this analyzed scenario, the wide age range found in the under-13 category motivated this research. Since coaches face the challenge of organizing training for a broader age range, the possibility of organizing training based on maturational stages could be crucial for better player performance. However, when maturational status was analyzed, no players were identified in the post-PHV stage, and this condition result in non-significant differences in the variables compared to the games organized by chronological age.

20. ICC values seem too low (e.g. for TRIMP, technical parameters). I don’t think they could be averaged in this way.

**RESPONSE:** 20. ICC values seem too low (e.g. for TRIMP, technical parameters). I don’t think they could be averaged in this way.

RESPONSE: Thanks for this comment. The ICC value for technical parameters was low especially because of the open nature of the game (open skill) which is quite unpredictable, especially for the technical parameters. Thus, a high variability may indicate inconsistency in the technical parameters of young players. (Clemente et al. 2019) .

In any case, it is important to note that the analyses were not conducted using the average of the two games in each condition (BIO vs. CA). On the contrary, the linear-mixed models considered all available information for the games played under each condition. This was described in the text as follow:

Each participant had four observations in the models (i.e., two SSG played in the CA condition and two SSG played in their respective BIO condition).

For a better understanding and to avoid possible doubts about the statistical analysis of the data, we decided to remove the ICC of the analyzed variables from the text.

21. CMJ did not differ between PHV groups (even more, pre-PHV players achieved higher 17. performance than PHV players) indicating that maturation had no effects on performance. Why then we should expect differences in technical variables or player load?

**RESPONSE:** Thanks for this comment. The CMJ values were not statistically higher in pre-PHV than in PHV players, and these values help explain why there was no statistical difference between the groups in BIO. Since, although the maturational level was higher in the PHV group, the lower body strength/power levels were not statistically different.

22. Ln244-247. Description of the results seem confusing. In line 245 the increase of 11.5% in CA condition compared to BIO condition refer to the pre-PHV group (as stated in the manuscript) or to the PHV group? (as illustrated in figure 1). Please clarify and correct accordingly.

**RESPONSE:** Thank you for this comment. We recognize that there was an error regarding the percentage value relating to the increase in PlayerLoad, so the text has been corrected.

*“for the pre-PHV players the CA condition led to 11.5% higher PlayerLoad*”

23. Ln 253-254. Similarly to previous comment, please check.

**RESPONSE:** Thank you for this comment. Thank you for this comment. The text was corrected, in fact the reported value was different from the values presented in the results section, so the text was rewritten as follows:

*“A main novelty of the present study was the higher external training load (average increase of 11.5%) experienced by the pre-PHV players when playing in the bio-banded SSG”*

Discussion
24. Discussion is well written and easy to follow. What may be a possible explanation for the increase in external load for the pre-PHV group under biobanding grouping? Biobanding however, seems less effective for the players in PHV group, this result is not discussed in the manuscript, and I think it deserves more attention.

**RESPONSE:** Thank you for this comment. We agree with the reviewer, and it was added in the text as follow:

*This condition can be partly explained by the age range of the players evaluated, as older players, when playing according to chronological age, were possibly more exposed to the demands of the game compared to the younger athletes.*

25. Ln. 294. Please check for grammar and consider revising (…PlayerLoad obtained of pre-PHV….)

**RESPONSE:** Thank you for this comment. This sentence was rewritten as follow:

*The BIO condition differently impacted the PlayerLoad obtained of pre-PHV and PHV players*

26. Ln315-316- Please check for grammar and consider revising.

**RESPONSE:** Thanks for this comment. The grammar was reviewed.

27. Ln340. Format of the in-text citation!!! (Romann et al., 2014).

**RESPONSE:** Thanks for this comment. It was corrected.

28. Ln356. Exactly, this is what I meant earlier, all players were under 13 years, having therefore few (if any) differences in maturation. The design here included players of differences in CA, but not definitely in maturation. I think a reverse design would be more useful, that is players of the same CA, but of distinct maturational status.

**RESPONSE:** Thanks for this comment. In fact, there was significant difference in maturation level (-2.2 vs. -0.89). It is possible to make this design reverse, however, the aim of this study was to verify the effect of the biobanding in this context, so, we think that the design is adequate and, in some way, it can contribute to the understanding of the use of biobanding in categories whose players have not yet reached PHV.

29. References are not formatted according to the journal’s instructions. Please check and correct accordingly.

**RESPONSE:** Thanks for this comment. We corrected all the references and citations.

30. Please mark significant differences in figure 1.

**RESPONSE:** Thanks for this comment. The significance of figure 1 is shown in table 4.

1. *doi: 10.1111/sms.13889*
2. *doi: 10.1123/pes.25.2.186*
3. *doi: 10.1371/journal.pone.0127505*
4. *doi: 10.5628/rpcd.23.03.43*
5. *doi: 10.1519/JSC.0000000000004202*
6. *doi: 10.3390/jfmk6040090*
7. *doi: 10.1519/JSC.0b013e31822a61cf*
8. [*doi*](http://dx.doi)*: 10.1123/pes.2013-0096*
9. *doi: 10.1519/JSC.0000000000004187*
