# Supplementary material for: Effects of biobanding on training loads and technical performance of young football players
Source: PLoS One. 2025 Feb 13;20(2):e0317432. doi: 10.1371/journal.pone.0317432 (PMC11824955; doi:10.1371/journal.pone.0317432)
Supplement: Attachment — Submitted filename: Response Letter_PlosOne-round 2_post AM4 (1).docx [file pone.0317432.s002.docx]

October 29, 2024

RE: Revised version of the manuscript - PONE-D-24-11339 “EFFECTS OF BIOBANDING ON TRAINING LOADS AND TECHNICAL PERFORMANCE OF YOUNG FOOTBALL PLAYERS”.

PLOS ONE”

Dear Editor-in-Chief,

First of all, we appreciate the time devoted to evaluating our manuscript, helping us to craft hopefully an improved version. We are pleased to clarify your concerns, which we believe will improve the impact and quality of our work. We have made a concerted attempt to systematically address the specific concerns raised for this revision. We have highlighted the alterations in the manuscript using the track changes tool. Your constructive and insightful points have improved selected areas of the manuscript significantly. We believe that the manuscript has been significantly improved based on your insightful comments and we hope that based on these modifications, the manuscript is now closer to acceptance.

**Reviewer Comments:**

**Reviewer #1:**

1. Before recruiting participants, did the authors calculate the minimum sample size required for the study? Please report the specific calculation method and results.

**RESPONSE:** No a priori sample size calculation was done. We investigated the sport club we had access to, in which there were 20 children playing in the team. We proposed the research for the team staff, they agreed to participate, and all children were involved. Unfortunately, having a limited number of participants is a limitation when working in a real-life setting inside a sports club.

Furthermore, it is worth highlighting that performing power calculation for linear mixed model (LMM) is not a trivial task (please see: https://doi.org/10.3758/s13428-021-01546-0). The choice for a LMM approach is that this analysis is able to model the structure of the data (repeated measures) and provide estimates considering variability within/between subjects. 20 participants with four observations each allows the model to tease apart the effects of the experimental design on our dependent variables.

If this reviewer is aware of any a posteriori sample size calculations and, most importantly, how the 20 participants in our sample limits the power in our analysis, we are happy to follow and proceed accordingly.

In the manuscript we have acknowledged in the limitation that the results are limited to a small sample size with future larger investigations needed to strengthen the biobanding hypothesis.

2. In the Statistical analysis section, did the authors analyze the independence, distribution, and variance of all indicator data before using the T-test? It is necessary to report whether the sample data meet the assumptions required for the use of the independent samples T-test and Mixed model linear regression methods before using them. If some indicators in the study do not apply to the above methods (such as non-linearity, not meeting the normal distribution), it will lead to unreliable research results and conclusions, and other methods should be considered for analysis. It may be inappropriate to present data in the form of mean and standard deviation before knowing the distribution of the data. From the data submitted by the authors, some data seem not to conform to the normal distribution and homogeneity of variance assumptions.

**RESPONSE:** We thank the reviewer for the careful assessment of our article. For the comparisons between groups presented in Table 2, we have now checked for normality of distribution and homogeneity of variances. In cases when not met, we applied non-parametric tests (please see our answer to question #5 below). It is worth noting that non-parametric statistics let to virtually exact the same results and interpretation.

For the LLMs (and GLLMs see below). Our data structure contains dependent data points (e.g., same participant observed in different moments) and the assumption of independence is not met. Hence why we have used linear mixed models, which accounts for the repeated nature of our investigation (e.g., same participants with 4 data points in all analysis). Moreover, given the predictors (independent variables) are binary (either one group/condition or the other) the coefficients will always meet the assumption of linearity.

Regarding the normal distribution, it is paramount to stress that for (G)LLMs the distribution of the dependent variable and homogeneity of variances are not assumptions to be met (please see relevant chapters in: *1 -"Discovering statistics Using r by Andy Field, Jeremy Miles, and Zoë Field; 2012"* for a very basic introduction*; and 2 - "Linear Mixed-Effects Models Using R. A Step-by-Step by Approach by Andrzej Gałecki and Tomasz Burzykowski"* for a more detailed discussion).

However, as per reviewer's suggestion, for the LLMs we have also assessed normality for the training load variables using the Shapiro wilk test for Session-RPE (P = 0.002), player load total (P = 0.15) and TRIMP (P = 0.14). As observed, Session-RPE did not follow a normal distribution which did not alter the normal distribution of the residuals. Please see below visual analysis and Shapiro Wilk's results in parenthesis.

**Trimp residual distribution (W = 0.98061, p-value = 0.2656).**

**Session-RPE residual distribution (W = 0.98622, p-value = 0.5486).**

**Player load total (W = 0.97953, p-value = 0.2281).**

Regarding the homogeneity of the variances (heteroscedasticity). Although not an assumption, this was checked looking at the fitted vs the predicted plots from the regression. Please see below for the models of training load:

**Trimp model:**

**Session-RPE model:**

**Player load model:**

Although a tendency seems to exist for TRIMP, this is not affecting the models. To test that, we inserted random effects for the intercept and slopes (to model the possible impact of heteroscedasticity). All model statistics (bayes factor, AIC, BIC, and p values) favor the model with only random intercepts, and models led to the same predictions (please also note the linearity) as follows:

**Session-RPE**

**Playerload**

**TRIMP (more about TRIMP on answer to #3)**

Data in figures are predictions of the dependent variable by condition (CA and BIO) split by maturation status. Red line: model with random intercepts and slopes; Green line: model with random intercepts only.

Finally, technical performance variables used in the investigation (total of passes, shots, etc). These variables are originally summed count data (e.g., number of complete passes summed by the number of incomplete passes). Importantly, technical actions do not follow a normal distribution, but rather a Poisson distribution, in some cases with the presence of zero inflated values:

**Total tackle:**

**Total steals:**

**Total Shots:**

In addition to the 0-inflated characteristic, we also agree with the reviewer (answer #3) that reliability of the technical actions is an issue. Furthermore, given the open nature of the SSGs reliability of all technical actions were very low (please see our answer to question #3). For this, in accordance with the reviewer, we decided not to test the individual technical variables and rather decided to only analyze involvement (sum of every technical action in the SSGs) the data.

Involvement was analyzed using a Linear Mixed Models, which failed to meet the assumptions after being log transformed:

**Involvement:**

**Residuals involvement (W = 0.9702, p-value = 0.05848)**

Due to this, we have also analyzed involvement with a mixed model generalized linear model (GLMM) with a Poisson distribution:

**Left linear mixed model/right Poisson GLMM**

|  | **Involvement** | | | | | |
| --- | --- | --- | --- | --- | --- | --- |
| *Predictors* | *Estimates* | *CI* | *p* | *Incidence Rate Ratios* | *CI* | *p* |
| (Intercept) | 3.18 | 2.96 – 3.41 | **<0.001** | 25.46 | 20.55 – 31.53 | **<0.001** |
| cond [IC] | -0.15 | -0.46 – 0.16 | 0.350 | 0.89 | 0.66 – 1.20 | 0.442 |
| st [PVC] | -0.01 | -0.33 – 0.31 | 0.952 | 1.00 | 0.74 – 1.35 | 0.994 |
| cond [IC] × st [PVC] | 0.20 | -0.24 – 0.63 | 0.379 | 1.18 | 0.77 – 1.81 | 0.445 |
| **Random Effects** | | | | | | |
| σ^2^ | 0.24 | | | 0.04 | | |
| τ_00_ | 0.01 _id_ | | | 0.10 _id_ | | |
| τ_11_ |  | | | 0.20 _id.condIC_ | | |
| ρ_01_ |  | | | -0.70 _id_ | | |
| ICC | 0.04 | | | 0.72 | | |
| N | 20 _id_ | | | 20 _id_ | | |
| Observations | 80 | | | 80 | | |
| Marginal R^2^ / Conditional R^2^ | 0.019 / 0.058 | | | 0.027 / 0.725 | | |

Looking at the table above at the Poisson model, contains a random slope and the model was favored by the AIC and BIC statistics.

Given all the above, and considering the reviewer’s suggestions, in the new version:

Table 2 has been adjusted to include comparisons using non-parametric Mann Whitney test in cases when normality of distribution was not met. Furthermore, data in table was triple checked and now contains the right values (please see our answer to your question #5).

We have deleted the technical performances from the analysis and only included involvement and a variable to indicate overall participation in the SSGs matches. Also, involvement was analyzed using a Poisson GLLMs with a random intercept for participants to account for the repeated (independence) nature of our dataset and a random slope which improved significantly the prediction of the model. This information has been included in the statistical analysis section.

We have also included in the discussion a paragraph discussing the poor reliability of the technical data and how this may impact interpretation (please see our answer to #3).

3. Are the ICC values reported in lines 231-233 the ICC values between the individual internal tests of the participants? If possible, please report the ICC and CV values for all test indicators to enhance the reliability of the results. In addition, the ICC values for session-RPE, TRIMP, and technical variables (each indicator's ICC should be reported specifically) are below the generally acceptable minimum standard in research (ICC > 0.75 and CV < 15%) [1-4]. From the submitted data, it appears that there is a large difference in data for the same athlete under the same conditions in two competitions.

[1]Baumgartner TA, Jackson AS. Measurement for evaluation in physical education and exercise science.Meas Eval Phys Educ Exerc Sci. 1998. <https://doi.org/10.4324/9781315312736>.

[2]Baumgartner TA, Chung H. Confidence limits for intraclass reliability coefficients. Meas Phys Educ Exerc Sci 5: 179–188, 2001.

[3]Safrit, M. J., & Wood, T. M. (1995). Introduction to measurement in physical education and exercise science (3rd ed.). New York: Mosby.

[4]Atkinson G, Nevill AM. Statistical methods for assessing measurement error (reliability) in variables relevant to sports medicine. Sports Med. 1998;26(4):217-238. doi:10.2165/00007256-199826040-00002

**RESPONSE:** We thank the reviewer for the comments about reliability. However, the aim of our investigation is not to present the reliability of technical and training load variables during small-sided games (SSG). This is expected to occur given the open nature of training routines, specially SSGs. With the low reliability (high variability in the technical and training load performance during SSGs) in mind, we decided to use two training sessions per experimental manipulations. This was done to try and get closest to the true technical and physiological responses of the SSGs in children, and two similar SSG is also what is realistic for an experimental manipulation inside the training routine of the team.

However, we have now inserted matches (SSG #1 and SSG #2) as a fixed effect variable to test whether players changed the outcomes across matches. Random slopes were tested but they did not increase the model fit. This was done to check for changes in the outcome variable across the two repeated SSGs as one indicative of reliability (we did not expect the variables to change from SSGs to SSGs).

**The table below describes the effect of SSG matches on the outcome of the study.**

**Table reliability of the variables included in the manuscript. Models are LMMs**

|  | **TRIMP** | | | **PLOT** | | | **Session-RPE** | | |
| --- | --- | --- | --- | --- | --- | --- | --- | --- | --- |
| *Predictors* | *Estimates* | *CI* | *p* | *Estimates* | *CI* | *p* | *Estimates* | *CI* | *p* |
| Intercept | 107.88 | 101.95 – 113.81 | **<0.001** | 394.22 | 366.41 – 422.03 | **<0.001** | 5.35 | 4.63 – 6.07 | **<0.001** |
| jog2 | -15.72 | -23.– -8.12 | **<0.001** | -18.50 | -41.02 – 4.01 | 0.106 | 0.75 | -0.04 – 1.54 | 0.061 |
| condIC | -15.63 | -23.22 – -8.03 | **<0.001** | -11.63 | -34.15 – 10.88 | 0.307 | 0.05 | -0.74 – 0.84 | 0.899 |
| jog2:condIC | 22.06 | 11.31 – 32.80 | **<0.001** | 8.07 | -23.78 – 39.91 | 0.615 | -0.70 | -1.81 – 0.41 | 0.213 |
| **Random Effects** | | | | | | | | | |
| σ^2^ | 145.46 | | | 1276.91 | | | 1.55 | | |
| τ_00_ | 31.52 _id_ | | | 2618.92 _id_ | | | 1.03 _id_ | | |
| τ_11_ |  | | |  | | |  | | |
| ρ_01_ |  | | |  | | |  | | |
| ICC | 0.18 | | | 0.67 | | | 0.40 | | |
| N | 20 _id_ | | | 20 _id_ | | | 20 _id_ | | |
| Observations | 80 | | | 80 | | | 80 | | |
| Marginal R^2^ / Conditional R^2^ | 0.191 / 0.335 | | | 0.018 / 0.678 | | | 0.035 / 0.419 | | |
| log-Likelihood | -308.997 | | | -406.701 | | | -142.854 | | |

Reliability of the count technical variables included in the manuscript. Models are Poisson with a random intercept and a random slope

As clearly observed, the variable TRIMP changed across the SSGs with an interaction between condition and TRIMP.

Because of the changes observed for the TRIMP variable across matches, we have now adjusted the effects of condition for matches. This did not change the observed results.

We have now discussed the poor reliability is in the new version of the manuscript and inserted a paragraph with the findings of the changes in SSG #1 compared to SSG #2 (e.g., reliability). Importantly, the ICC used in the analysis and article was obtained from the (G)LMMs. This is stronger than using separated analysis as (G)LMMs consider the variability between conditions as well as within participants whilst accounting for the dependence in the observations.

Please see the results discussion section where we present the ICC values from the linear-mixed models and address the issue of poor reliability of the performance variables during SSGs.

4. Should Table 1 on line 228 be Table 2?

**RESPONSE:** Done accordingly.

5. The average value of each group in Table 2, multiplied by the number of people in each group and then added together, divided by the total number of people to get the average value (such as CMJ), has a significant difference from the overall average value in Table 2. This difference is greater than the general error of data processing, please ask the authors to confirm whether there may be calculation errors in the data. In addition, the data involved in this study (such as the original data of Age, yPHV, Height, Body mass, CMJ, etc., in Table 2) have not all been uploaded to the database, and the above data should be displayed in the database as part of the study.

**RESPONSE:** We have now carefully inspected the results of Table 2. We found the mistake as in Brazil commas are used to separate decimal places and when moving to the statistical software used to describe participants a mix of values separated either by commas or dots led to different descriptive averages. We have now reassessed the data and tripled checked all values presented. We can confirm the data now is correct and we have also uploaded it to the repository.

Furthermore, data in Table 2 was also assessed for normality (Shapiro Wilk) and homogeneity of variances (Levene test). In cases when normality of distribution (age, body mass, and cmj) and homogeneity of variances (age and body mass) was an issue, we used the Mann Whitney Rank test to compare the descriptive variables. This information has now been added to the manuscript and Table 2 corrected accordingly.

6. In the data uploaded by the authors, it was found that many athletes' data are not completely complete, such as in the data of steal_complete, steal_incomplete, tackle_incomplete, and other indicators, many athletes do not have data points, the lack of the above data points will affect the statistical power and significance, and to some extent affect the results and conclusions. Therefore, the actual number of data points for each indicator should be reported in the table (such as adding the number of data points for each indicator in Table 3). In addition, according to the general reporting standards, should the data such as Total shots (n) in Table 3 be reported to one decimal place like other data?

**RESPONSE:** We have adjusted the lack of data with zeros. Those missing values were there because in some situations athletes did not perform the technical parameter under assessment. We have now replaced missing by zeros and all analysis about technical performance were completed once again. Please see our answer to your question #2.

7. The specific statistical power, fixed-effects, and random effects test results should be reported in Tables 4 and 5 (or in the text).

**RESPONSE:** Fixed effects were already present in the Tables. We have now added the random as per reviewer's suggestion. We did not include the power as this is not something the models provide (please see: https://doi.org/10.3758/s13428-021-01546-0).

8. I believe that the number of tests conducted in your study is relatively small, and there is a large difference in the performance of the same subjects in different training and competitions within your research.

**RESPONSE:** We agree with the reviewer that including more SSGs across the experimental designs would increase our ability to draw conclusions about the effects of biobanding. However, as discussed in our previous answer, the only variable that changed across SSGs was TRIMP and some technical performances measured. This information has been included in the manuscript (see statistical section and discussion). Apart from TRIMP and isolated technical variables (included in the analysis as involvement) we argue that there was not a large difference in athlete’s performance. Indeed, as argued we expected training load and technical performance to vary between SSGs given the poor reliability of these outcomes in a real-world training session.

Therefore, the results of the two tests may not adequately represent the true athletic performance of the athletes. Additionally, factors such as the physical condition of the athletes, their relative positions on the field, and the level of defense against the subjects may vary across different tests, and these factors can significantly affect the subjects' performance. The authors did not control for these factors in their study, so the results may not support the authors' conclusions. It is recommended to increase the number of tests to better understand the true conditions of the subjects, and to control the impact of the aforementioned factors on the results.

**RESPONSE:** We agree with the reviewer that including more SSGs situations across the experimental designs would increase our ability to draw conclusions about the effects of biobanding. Future investigations should use more SSGs and also use different experimental designs (e.g., one club uses CA and other club uses biobanding throughout a whole training season). This would also allow to test the impact of physical fitness and playing position (e.g., which athletes benefit more across the year and what is the impact of the initial fitness levels?). However, this was not the aim of our study which simply aimed to investigate whether biobanding has any effect on training load during SSGs, building on previous work that either demonstrate benefits of biobanding or hypothesized that benefits may exist.

Finally, we would like to add that our conclusions are supported by our results. The results show that Biobanding influences external training loads of young athletes during SSGs. We stress that the conclusions are robust and even after a thorough review and the application of different analysis presented in this rebuttal (see answer to your question #2), results still show a significant effect of BIO on PlayerLoad.

**Reviewer #2:** I would like to thank the authors for their revision considering almost all previous comments. A convincing explanation of the age range examined in this study seems to be still missing, however, this does not significantly affect the manuscript's quality. The common practice to group athletes of 10-13 years in one age-group (as stated by the authors) seems to induce unbalanced training loading, based on the results the authors could consider including clear recommendations for policy makers and club administration for a more effective grouping method.

**RESPONSE:** Thank you for this suggestion. We have now inserted the practical application of the results in the last paragraph as follow:

PRATICAL APPLICATIONS

The changes observed in the present investigation suggest that exploring biobanding may be an innovative opportunity for coaches to effectively tailor training sessions, particularly in age groups below the PHV. By grouping athletes based on their biological maturity rather than chronological age, coaches gain a significant tool to better organize training sessions. This approach acknowledges the diverse developmental stages of young athletes, allowing for more targeted and appropriate training, maximizing growth and skill development, especially when there is an early talent promotion process within youth development categories. An important practical application of biobanding is that coaches can conduct maturational band training sessions when they aim to equalize external training loads. An important practical application of biobanding is that coaches can conduct maturational band training sessions when they want to equalize external training loads.
